# Gender and Age Differences in Hourly and Daily Patterns of Sedentary Time in Older Adults Living in Retirement Communities

**DOI:** 10.1371/journal.pone.0136161

**Published:** 2015-08-21

**Authors:** John Bellettiere, Jordan A. Carlson, Dori Rosenberg, Anant Singhania, Loki Natarajan, Vincent Berardi, Andrea Z. LaCroix, Dorothy D. Sears, Kevin Moran, Katie Crist, Jacqueline Kerr

**Affiliations:** 1 San Diego State University/University of California, San Diego Joint Doctoral Program in Public Health (Epidemiology), University of California San Diego, La Jolla, California, United States of America; 2 Center for Behavioral Epidemiology and Community Health, Graduate School of Public Health, San Diego State University, San Diego, California, United States of America; 3 Center for Children's Healthy Lifestyles and Nutrition, Children’s Mercy Hospital, Kansas City, Missouri, United States of America; 4 Department of Health Services, University of Washington School of Public Health, Seattle, Washington, United States of America; 5 Department of Family Medicine and Public Health, University of California San Diego, La Jolla, California, United States of America; 6 Computational Science Research Center, San Diego State University, San Diego, California; School of Public Health of University of São Paulo, BRAZIL

## Abstract

**Background:**

Total sedentary time varies across population groups with important health consequences. Patterns of sedentary time accumulation may vary and have differential health risks. The purpose of this study is to describe sedentary patterns of older adults living in retirement communities and illustrate gender and age differences in those patterns.

**Methods:**

Baseline accelerometer data from 307 men and women (mean age = 84±6 years) who wore ActiGraph GT3X+ accelerometers for ≥ 4 days as part of a physical activity intervention were classified into bouts of sedentary time (<100 counts per minute). Linear mixed models were used to account for intra-person and site-level clustering. Daily and hourly summaries were examined in mutually non-exclusive bouts of sedentary time that were 1+, 5+, 10+, 20+, 30+, 40+, 50+, 60+, 90+ and 120+ minutes in duration. Variations by time of day, age and gender were explored.

**Results:**

Men accumulated more sedentary time than women in 1+, 5+, 10+, 20+, 30+, 40+, 50+ and 60+ minute bouts; the largest gender-differences were observed in 10+ and 20+ minute bouts. Age was positively associated with sedentary time, but only in bouts of 10+, 20+, 30+, and 40+ minutes. Women had more daily 1+ minute sedentary bouts than men (71.8 vs. 65.2), indicating they break up sedentary time more often. For men and women, a greater proportion of time was spent being sedentary during later hours of the day than earlier. Gender differences in intra-day sedentary time were observed during morning hours with women accumulating less sedentary time overall and having more 1+ minute bouts.

**Conclusions:**

Patterns identified using bouts of sedentary time revealed gender and age differences in the way in which sedentary time was accumulated by older adults in retirement communities. Awareness of these patterns can help interventionists better target sedentary time and may aid in the identification of health risks associated with sedentary behavior. Future studies should investigate the impact of patterns of sedentary time on healthy aging, disease, and mortality.

## Introduction

Sedentary behavior increases the risk of diabetes, cardiovascular disease, and death [1–3]. The negative consequences are independent of physical activity [4]. This evidence has prompted national guidelines in Australia and the United Kingdom (UK) to encourage individuals to reduce sedentary time (i.e., sitting) [5,6].

Most research on the deleterious effects of sedentary behavior comes from self-reported measures of sitting behavior (eg, time spent watching TV, time spent on the computer) [1]. Objective measures of activity are now being used to advance our understanding of sedentary behavior, most recently in older adults [7]. For example, we know that a nationally representative sample of U.S. adults over age 60 spend the most time being sedentary [8,9], and that men are more often sedentary (8.7 hours per day) than women (8.3 hours per day) [9].

Most studies analyzing objectively measured data quantify sedentary time as a single measure, most commonly as the total number of minutes per day spent sedentary. However, this method does not take into account patterns of sedentary behavior that may have important health consequences. For example, studies have shown that breaks from sitting time have stronger associations with physical functioning among older adults than total sedentary time [10,11]. In adult populations, laboratory studies have demonstrated that unbroken bouts of sitting for at least 20 minutes results in acute changes in post-meal metabolism [12,13], suggesting that the way sedentary time is accumulated may have important health consequences.

Despite the importance of patterns (i.e., how sedentary time is accumulated) of sedentary time, few studies have described detailed patterns among older adults and, to our knowledge, no studies have reported patterns for U.S. adults living in retirement communities. One study found that older men from a population-based cohort in the UK (mean age = 78 years) [14] were sedentary an average of 10.3 hours per day. Their sedentary time was accumulated on average in 72 bouts per day with more than 56% of sedentary time accumulated in bouts ≥ 20 minutes [14]. Another study used data from 7,247 women participating in the Women’s Health Study (mean age = 71 years). Researchers observed a mean sedentary time of 9.7 hours per day accumulated in 86 bouts; 44% of sedentary time was accumulated in bouts ≥ 20 minutes [15]. Both studies showed that patterns of sedentary behavior varied with age with the oldest adults having the smallest number of sedentary bouts and spending the most time being sedentary. Comparisons of data across the two studies suggest that men and women accumulate sedentary time differently but the heterogeneity of the two populations make direct comparisons difficult. Furthermore, neither of the above mentioned studies report intra-day patterns of sedentary activity, information which might have important applications for identifying appropriate and effective targets to intervene on prolonged sitting [16].

The present study describes detailed daily and hourly patterns of objectively measured sedentary time of older men and women living in retirement communities. The scant evidence comparing age-related health between older adults living in general vs. retirement communities suggests the two populations are different, with retirement community residents having worse agility, balance, fear of falling, and fall histories [17,18]. Older adults living in retirement communities are also at higher risk for sedentary behavior and for accumulating sedentary time in long (≥ 30 minute) bouts [19] making this an important population for health interventions. With a larger proportion of both men and women in U.S. population reaching retirement age (65 years) [20], residency in retirement communities is expected to increase. Thus, a better understanding of how sedentary time is accumulated in this population, including age and gender differences, will inform health promotion efforts.

## Methods

### Participants

Participants were men and women living in continuing care retirement communities (CCRCs) which often are campus-like communities with independent living, assisted living, and skilled nursing care available on site. All participants in this study were in independent living situations when they participated in baseline measures of a site-randomized controlled trial (Multilevel Intervention for Physical Activity in Retirement Communities; MIPARC) [21]. Of twenty one sites that met eligibility requirements (i.e., > 100 residents, offered independent living accommodations, < 1 mile from a park or shopping), six sites did not respond when contacted, two sites declined to participant, and two sites did not have enough eligible participants; in all, eleven sites consented and participated. To ensure participants were able to provide informed consent, participate in intervention components, and to reduce the risk of falls and injury due to the physical activity intervention, residents within each site were eligible if they met the following criteria: ≥ 65 years of age; spoke/read English; could complete written assessments and hold a conversation over the phone; available to attend weekly meetings; no falls in the previous 12 months resulting in hospitalization; could walk 20 meters without physical assistance; and could complete the Timed Up & Go [22] test of mobility in less than 30 seconds. From the participating sites, 478 participants were screened; 56 were ineligible and 115 declined to participate. Data for those who declined to participate were not available. Of the 56 that were ineligible, 37 (66%) were excluded due to a previous fall, 6 (11%) due to vision problems, and the remaining 13 due to poor cognitive function (n = 2), failure of the Timed Up & Go test (n = 2), not having the approval of a physician (n = 2), not being able to attend meetings (n = 2), being hospitalized (n = 1), moving within the study period (n = 1), being too young (n = 1), or for other reasons (n = 2). Those who were ineligible did not differ by age or gender from eligible participants.

### Data collection

The research protocol was reviewed and approved by the institutional review board within the University of California, San Diego, Human Research Protections Program (#091028). Study staff obtained written consent from participants following a short introduction to the study. Eligible participants were asked to complete questionnaires gathering demographic information as well as measures of cognitive, mental, and physical health. They were given hip-worn accelerometers to wear during waking hours for 1 week. Participants were instructed to remove the devices before showering, swimming or sleeping. Participants were called one to two times during the wear week to prompt compliance. One week later, participants returned the accelerometers which were immediately screened to ensure sufficient data were captured. Wear time criteria were considered met if there was at least 4 days of 600+ minutes or 2400 total minutes across 4 days. Participants were asked to re-wear the accelerometers if these criteria were not met.

### Accelerometer data processing

ActiGraph GT3X+ accelerometers (ActiGraph, LLC; Pensacola, FL) were set to sample acceleration at 30 Hz using the low frequency extension. Data from devices were first processed using ActiLife software v6.3 to convert data to 1-minute epochs. An improved algorithm for identifying non-wear time was used to enhance accuracy of estimates of time spent sedentary; [23] the algorithm has been used in large epidemiology cohorts [14,15]. The algorithm flags periods with ≥90 consecutive minutes of zero counts on the vertical axis as non-wear time; to allow for movement of the unworn device, two minutes with movement (counts > 0) were permitted as long as ≥30 minutes of non-movement were observed before and after it [23]. Each day was then classified as valid (or not) if at least 10 hours of wear time was recorded or if it was part of the 2400 total minutes.

### Measures of sedentary time

Each 1-minute epoch was classified as sedentary if it met the conventional cutpoint of <100 counts per minute (cpm) [8]. The GT3X+ is a tri-axis accelerometer, yet we used data from the vertical axis because the 100 cpm cut point was validated using only the vertical axis [8] and so our results could be compared with those from similar studies [14,15]. Bouts were defined as consecutive sedentary minutes. The *number of* and *time spent in* sedentary bouts of different durations as well as the longest-, median- and usual-bout length were then used to describe patterns of sedentary time. Bouts with durations ≥ 1, 5, 10, 20, 30, 40, 50, 60, 90, and 120 minute(s) were examined so results were comparable to other studies of older adults [14,15] and because there are no established thresholds for discriminating patterns of sedentary time that might impact health. Bout groupings were mutually non-exclusive, for example, time spent in 5+ minute bouts is a subset of time spent in 1+ minute bouts.

#### Number of sedentary bouts

For each bout duration, sedentary bouts were counted for each person-day.

#### Sedentary time in bouts

The total number of sedentary minutes per day spent in bouts of various durations was computed for each person-day.

#### Longest bout length

The longest bout length was used to describe the longest period of consecutive sedentary minutes across wear days. It was computed for each person-day by arranging bouts from smallest to largest across valid wear days and taking the maximum value.

#### Median bout length

The median bout length was computed for each person by arranging bouts from smallest to largest across all valid wear days and computing the midpoint.

#### Usual bout length

The usual bout length describes the bout duration at which half of total sedentary *time* is accumulated. Where median bout length describes the central tendency of bout *lengths*, the usual bout length describes the central tendency of *time* spent in sedentary bouts. As described in Stephens et al. (2014), usual bout length can be extracted from the equation $y(\varphi )\;=\;\frac{\varphi^{n}}{\varphi^{n}+\;W_{50}^{n}},\;$ where *y* is the cumulative proportion of total time accounted for by bouts of duration ≤ *φ*, *W*_50_ is the usual bout duration, and *n* is a free parameter [24,25]. This equation was estimated using nonlinear least-squares regression at the person level.

### Potential confounders

Physical and cognitive functioning as well as depression were measured as follows:

#### Physical functioning

The short physical performance battery (SPPB) assessed physical functioning using standard protocol which consisted of a test for balance, gait speed, and chair stands [26]. Scores of 0–4 were given for each test and the sum of scores across all tests formed the overall SPPB score [27]. Overall scores could range from 0 to 12 with higher scores indicating higher physical functioning.

#### Cognitive functioning

Participants completed the Trail Making Test part A then part B; the number of seconds needed to complete each test were used to measure cognitive functioning. Higher values indicate poorer performance on tests and therefore indicate worse cognitive functioning.

#### Depressive symptoms

Depressive symptoms were measured by summing responses to the self-administered 10-item Center for Epidemiologic Studies Short Depression Scale (CES-D 10) [28]. Scores could range from 0 to 30 with higher scores indicating higher levels of depressive symptoms.

### Statistical analysis

Sample characteristics were summarized with means and standard deviations for continuous measures and with percentages for categorical variables. Daily patterns and hourly patterns of sedentary time were examined separately.

#### Daily patterns of sedentary time

The median length and usual length of bouts were summarized across days for each person using linear mixed models with site entered as a random effect to account for the site-clustered sampling design. Means and standard errors of the number of bouts, time spent in bouts, and the longest bout length were estimated with person-day data using linear mixed models with site and participant entered as random effects to account for days nested within people and people nested within sites. Linear mixed models are preferred over traditional linear models to account for within- and between-level variability. They are especially important when analyzing nested data which violates assumptions of independence (e.g., because days within individuals and individuals within sites are non-independent) so linear regression cannot be used [29]. Age and gender were included in the mixed models to assess differences in sedentary measures associated with these factors. Age was categorized into three groups (65–79, 80–89, and >90) and *p*-values for age were reported for the omnibus categorical effect. To account for differences in accelerometer wear time across days and participants, we included daily accelerometer wear time in all models as a covariate. Potential confounding by physical functioning, cognitive functioning, and depression was accounted for by including SPPB scores, the results of Trail Making Tests A & B, and CES-D 10 scores in all models. Age-by-gender interactions were assessed for all sedentary measures. Alpha levels were set to 0.05.

#### Hourly patterns of sedentary time

For each participant, the number of 1+, 5+, 10+, 20+, 30+, 40+, 50+, and 60+ minute sedentary bouts *started* and minutes spent in 1+, 5+, 10+, 20+, 30+, 40+, 50+, and 60+ minute sedentary bouts were summarized for each hour (e.g., from 10:00 to 10:59) of valid days. Hours with less than 60 minutes of accelerometer wear time were excluded, resulting in the removal of 14.8% of hourly records. Mean hourly sedentary minutes were estimated for men and women separately using linear mixed models with site and participant entered as random effects to account for clustering of days within people and people nested within sites. SPPB scores, results of Trail Making Tests A & B, and CES-D 10 scores were included in all models to account for potential confounding. Participants were instructed to remove accelerometers to sleep and there were too few observations between the hours of 23:00 and 05:59, therefore this period of time was excluded from analyses. Values for all sedentary measures were plotted by gender over hour of the day and fit with Loess curves overlaid with 95% confidence bands. Significant hourly differences between men and women were determined as hours with non-overlapping confidence bands.

## Results

Participants were an average age of 83.6 (SD = 6.4) years (Table 1). Most were women (72%) and the majority had at least a college degree (64%). Fifty-nine percent of the sample were not married.

**Table 1:** Sample Characteristics.

	Mean (SD) or n (%)
Age	83.6 (6.4)
Age Categories	
67–79	83 (27%)
80–89	162 (53%)
> = 90	62 (20%)
Gender	
Female	222 (72%)
Male	85 (28%)
Education	
Less than College	106 (36%)
College or Above	192 (64%)
Marital Status	
Married	123 (41%)
Not Married	179 (59%)
Trail Making Test A (seconds)	54.4 (21.9)
Trail Making Test B (seconds)	134.2 (57.6)
Short Physical Performance Battery	8.6 (2.8)
Center for Epidemiologic Studies Short Depression Scale	5.5 (4.1)

### Number of Bouts

Participants wore accelerometer devices for an average of 13.5 (SD = 1.3) hours per day. Accelerometer wear time did not vary by gender. Differences in wear time by age were statistically significant; adults aged 65–79 years wore devices for 13.9 (SD = 1.4) hours, adults aged 80–89 years wore devices for 13.5 (SD = 1.3) hours, and adults aged ≥90 wore devices for 13.3 (SD = 1.3) hours. Wear time was a covariate in all models. Older adults accumulated an average 583.6 (se = 76.2) minutes of daily sedentary time in 70.6 (se = 13.7) bouts of at least 1 minute. Table 2 shows that women had significantly more 1+ minute bouts than did men while men had significantly more 10+, 20+, 30+, 40+, 50+, and 60+ minute bouts. The number of 5+, 90+, and 120+ minute bouts did not vary by gender. The number of 20+, 30+, and 40+minute sedentary bouts varied by age with the *oldest* participants (aged ≥90 years) having the most bouts. The number of 1+, 5+, 10+, 50+, 60+, 90+, and 120+ minute bouts did not significantly vary by age (p’s < 0.05).

**Table 2:** Number of Sedentary Bouts^a^, per day, mean(se).

			Gender					Age						
Bout Duration, minutes	Total^b^		Men^b^		Women^b^		p-value^c^	65–79^b^		80–89^b^		90+^b^		p-value^c^
1+	70.6	(13.7)	64.2	(1.9)	73.1	(1.4)	<0.001	71.1	(2.1)	70.9	(1.6)	69.1	(2.5)	0.558
5+	25.7	(3.7)	25.9	(0.6)	25.6	(0.4)	0.638	25.9	(0.6)	25.8	(0.5)	24.9	(0.7)	0.264
10+	15.0	(2.2)	15.9	(0.3)	14.7	(0.2)	<0.001	15.1	(0.3)	15.1	(0.2)	14.8	(0.4)	0.960
20+	7.8	(1.6)	8.8	(0.2)	7.4	(0.1)	<0.001	7.6	(0.2)	7.8	(0.2)	8.1	(0.3)	0.026
30+	4.9	(1.3)	5.6	(0.2)	4.6	(0.1)	<0.001	4.7	(0.2)	4.9	(0.1)	5.2	(0.2)	0.025
40+	3.2	(1.0)	3.8	(0.1)	3.0	(0.1)	<0.001	3.0	(0.1)	3.2	(0.1)	3.6	(0.2)	0.009
50+	2.2	(0.8)	2.7	(0.1)	2.1	(0.1)	<0.001	2.1	(0.1)	2.2	(0.1)	2.5	(0.1)	0.053
60+	1.5	(0.6)	1.8	(0.1)	1.4	(0.1)	<0.001	1.4	(0.1)	1.4	(0.1)	1.7	(0.1)	0.102
90+	0.4	(0.3)	0.4	(0.1)	0.4	(0)	0.212	0.3	(0.1)	0.4	(0)	0.4	(0.1)	0.395
120+	0.1	(0.1)	0.1	(0)	0.1	(0)	0.629	0.1	(0)	0.1	(0)	0.2	(0)	0.396

^a^ Variables computed using daily-level data.

^b^ Means are adjusted for accelerometer wear time, physical functioning, depression, cognitive functioning, as well as age and gender where appropriate.

^c^ p-values were derived from F tests following linear mixed models that regressed sedentary time on measures of age, gender, physical functioning, cognitive functioning, depression, and accelerometer wear time; Kenward-Roger approximations were used to estimate the denominator degrees of freedom.

### Time spent in bouts of varying lengths

Over half (57.5%) of sedentary time was accumulated in bouts of 20 minutes or longer (335.6 min./583.7 min.; see Table 3). Men accumulated an average 43.7 more minutes of total sedentary time that did women (615.1 min. vs. 571.4 min.; see Table 3). Sedentary time accumulated in bouts of 1+, 5+, 10+, 20+, 30+, 40+, 50+, and 60+ minutes was significantly higher among men with the greatest gender differences observed in time spent in 10+ and 20+ minute bouts, (66.1 and 67.6 minutes respectively). Time accumulated in 90+ and 120+ minute bouts did not vary by gender. Age was associated with time spent in sedentary bouts of 10+, 20+, 30+, and 40+ minutes with adults ≥90 years accumulating the most sedentary time, and adults 65–79 years of age spending the least amount of time in bouts of each duration. Sedentary time accumulated in 1+, 5+, 50+, 60+, 90+, and 120+ did not significantly vary by age.

**Table 3:** Minutes in Sedentary Bouts^a^, Per Day.

			Gender					Age						
Bout Duration, minutes	Total^b^		Men^b^		Women^b^		p-value	65–7 ^b^		80–89^b^		90+^b^		p-value^c^
1+	583.7	(76.2)	615.1	(9.7)	571.4	(6.4)	<0.001	575.0	(10.4)	583.2	(7.5)	596.6	(13.1)	0.091
5+	501.9	(82.7)	545.0	(10.3)	485.2	(6.3)	<0.001	491.4	(10.8)	501.5	(7.4)	515.9	(14.0)	0.080
10+	432.5	(85.2)	480.1	(10.7)	414.0	(6.6)	<0.001	420.5	(11.3)	431.5	(7.7)	450.1	(14.6)	0.047
20+	335.6	(88.5)	384.3	(11.3)	316.7	(7.0)	<0.001	319.6	(11.8)	334.0	(8.1)	359.9	(15.4)	0.016
30+	265.7	(84.2)	308.0	(10.9)	249.2	(6.7)	<0.001	250.6	(11.4)	263.1	(7.9)	291.4	(14.8)	0.022
40+	209.4	(74.5)	245.7	(10.0)	195.3	(6.2)	<0.001	193.9	(10.5)	206.7	(7.4)	235.3	(13.6)	0.018
50+	165.2	(66.2)	194.7	(9.1)	153.8	(5.7)	<0.001	154.0	(9.6)	161.4	(6.7)	189.1	(12.3)	0.059
60+	123.5	(59.1)	146.7	(8.8)	114.5	(6.0)	0.001	113.9	(9.2)	119.8	(6.8)	145.5	(11.5)	0.103
90+	44.0	(40.5)	49.9	(6.6)	41.7	(4.8)	0.235	39.5	(7.0)	43.5	(5.3)	51.3	(8.5)	0.315
120+	18.2	(23.0)	20.1	(3.9)	17.4	(2.5)	0.537	15.1	(4.0)	17.6	(2.9)	23.8	(5.2)	0.334

^a^ Variables computed using daily-level data.

^b^ Means are adjusted for accelerometer wear time, physical functioning, depression, cognitive functioning, as well as age and gender where appropriate.

^c^ p-values were derived from F tests following linear mixed models that regressed sedentary time on measures of age, gender, physical functioning, cognitive functioning, depression and accelerometer wear time; Kenward-Roger approximations were used to estimate the denominator degrees of freedom.

### Usual, longest, and median bout lengths

The observed usual bout length for the total sample was 17.0 minutes, indicating that participants spent 50% of their sedentary time in bouts of 17+ minutes (Table 4). The usual bout length varied by gender with men spending half of their sedentary time in longer bouts than women (p < 0.001). Median bout lengths across demographic categories ranged from 2.8 to 3.3 minutes and varied significantly by gender, but not by age. There were no significant age or gender differences in the longest bout length. Gender-by-age interactions were not observed for any of the sedentary bout variables.

**Table 4:** Sedentary Bout Characteristics.

		Longest Bout Length^a^, minutes			Median Bout Length^b^, minutes			Usual Bout Length^b^, minutes		
		mean^c^	se	*p*-value^d^	mean^c^	se	*p*-value^d^	mean^c^	se	*p*-value^d^
Total		81.9	(16.8)		2.9	(0.1)		17.0	(0.1)	
Age				0.476			0.581			0.142
	65–79	80.1	(2.7)		3.0	(0.1)		16.4	(0.7)	
	80–89	81.4	(2.0)		2.9	(0.1)		16.8	(0.5)	
	90+	85.4	(3.4)		2.8	(0.1)		18.4	(0.8)	
Gender				0.122			<0.001			<0.001
	Men	85.1	(2.6)		3.3	(0.1)		19.8	(0.6)	
	Women	80.6	(1.7)		2.8	(0.1)		16.0	(0.4)	

^a^ Variables computed using daily-level data.

^b^ Variables computed using person-level data.

^c^ Means are adjusted for accelerometer wear time, physical functioning, depression, cognitive functioning, as well as age and gender where appropriate.

^d^ p-values were derived from F tests following linear mixed models that regressed sedentary time on measures of age, gender, physical functioning, depression, cognitive functioning, and accelerometer wear time; Kenward-Roger approximations were used to estimate the denominator degrees of freedom.

**Fig 1:**
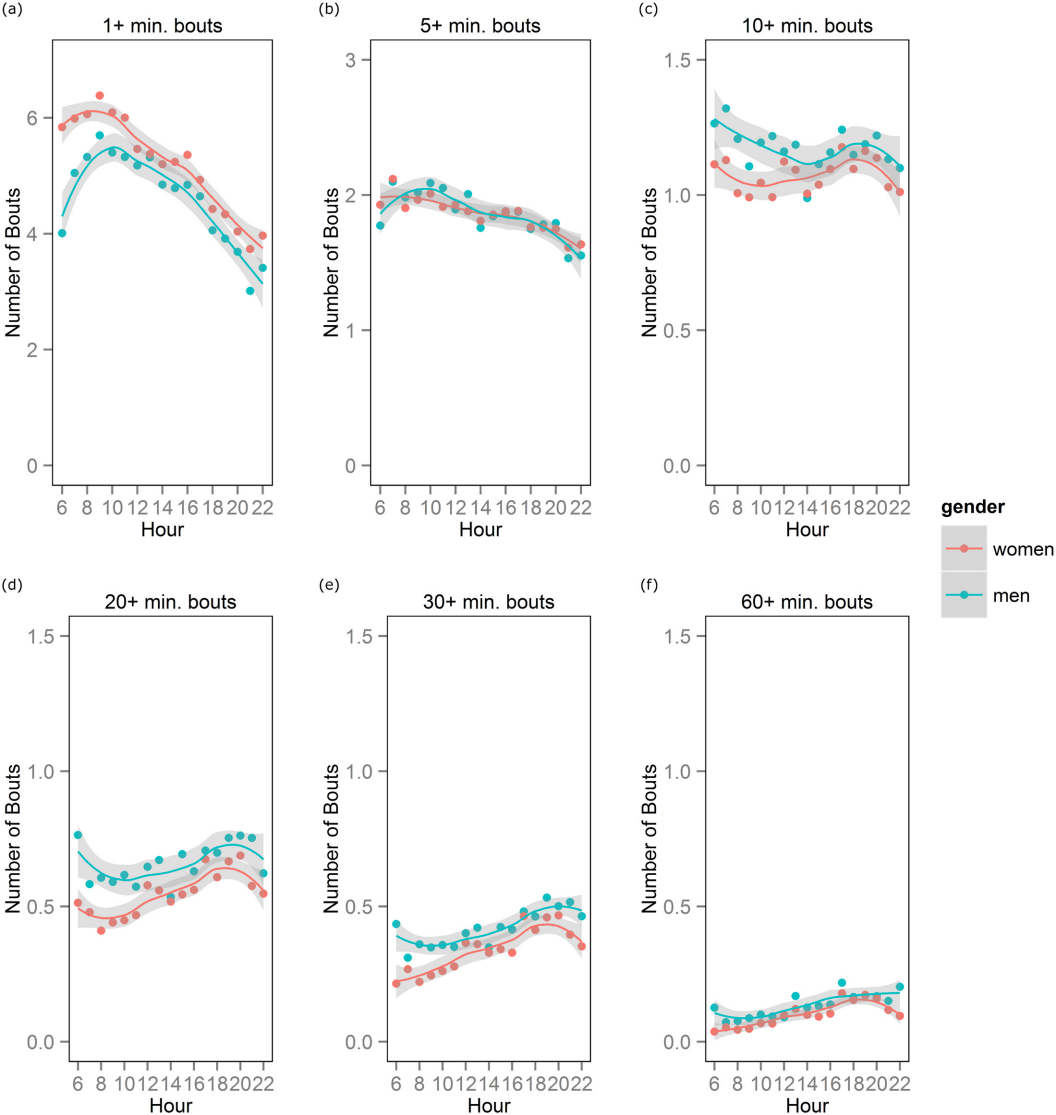
Number of sedentary bouts started during each one hour period between 06:00 to 22:59. The number of (a) 1+, (b) 5+, (c) 10+, (d) 20+, (e) 30+, and (f) 60+ minute bouts are plotted across hours of the day for men and women.

Fig 1 shows the number of bouts started during each 60 minute period beginning 06:00 to 06:59 and ending 22:00 to 22:59. During the 06:00 hour, men started an average of 4.0 bouts ≥1 minute in length while women started an average of 5.8 sedentary 1+ minute bouts. The number of 1+ min bouts started per hour varied significantly by gender at the 06:00, 07:00, 08:00, 09:00, and 10:00 hours with women having more bouts than men. The number of 5+ minute bouts did not significantly differ between men and women. Gender differences were observed in most early hours of the day for the number of sedentary bouts of 10+, 20+, and 30+ minutes with men having more bouts than women. Hourly trajectories for the number of sedentary bouts ≥ 40 and ≥ 50 minutes were similar to those of 60+ minute sedentary bouts and are shown in the appendix. With the following few exceptions, gender differences were not observed after the 12:00 hour for the number of bouts of all lengths: 1+ minute bouts for 19:00, 20:00, and 21:00 hours; 20+ minute bouts for the 20:00 hour; 30+ minute bouts for 20:00 and 21:00 hours; and 60+ minute bouts for the 22:00 hour.

**Fig 2:**
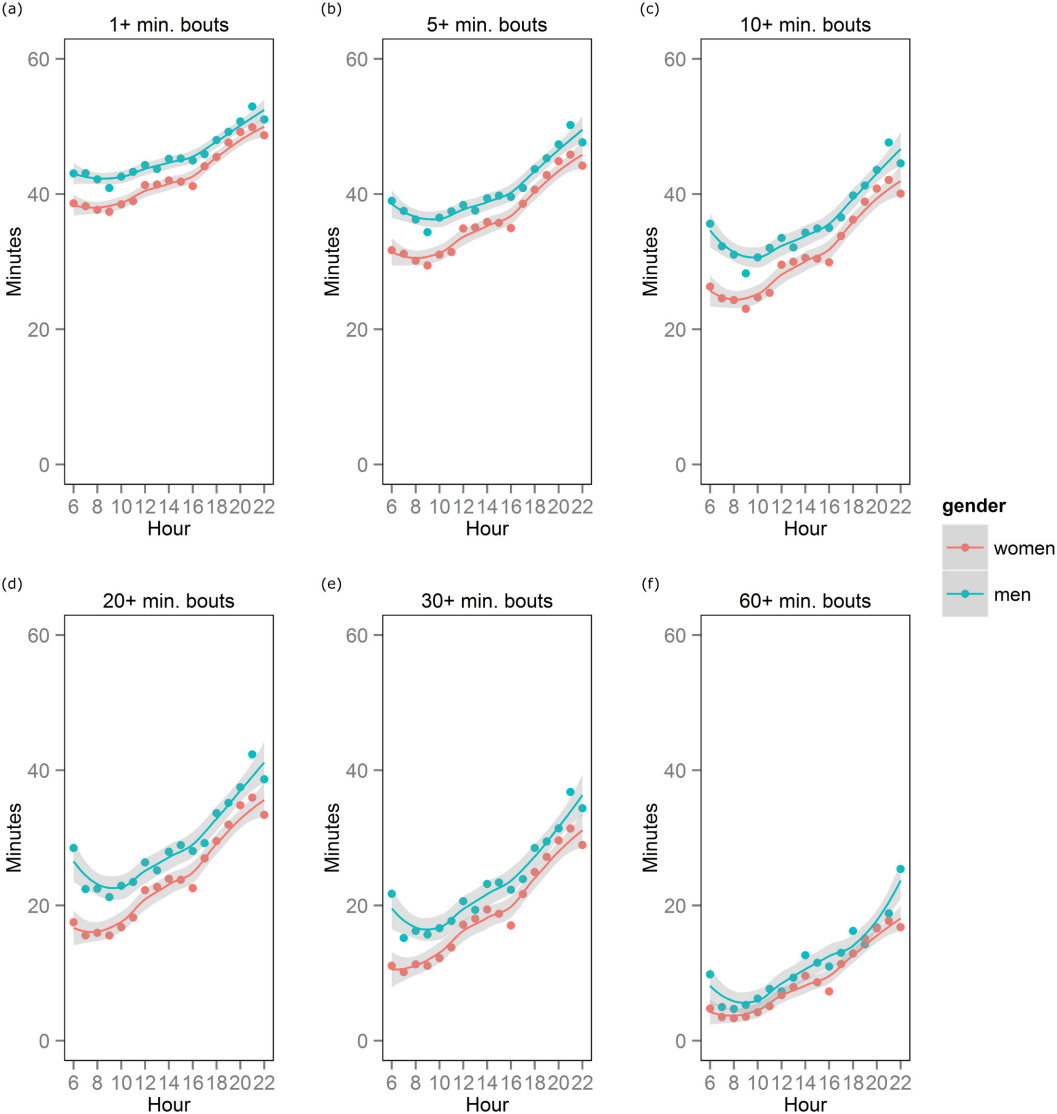
Sedentary minutes accumulated in bouts of various lengths for each one hour period between 06:00 and 22:59. The number of sedentary minutes spent in bouts of (a) 1+, (b) 5+, (c) 10+, (d) 20+, (e) 30+, and (f) 60+ minutes are plotted across hours of the day for men and women.

Fig 2 shows average sedentary minutes spent in bouts across various bout lengths for men and women. Overall, time spent in sedentary bouts decreased as minimum bout length increased; for example, during the 08:00 hour, men spent on average 42 minutes in 1+ minute bouts, 36 minutes in 5+ minute bouts, 31 minutes in 10+ minute bouts, 23 minutes in 20+ minute bouts, 16 minutes in 30+ minute bouts, and 5 minutes in 60+ minute bouts. Men accumulated more sedentary time than women in 1+, 5+, 10+ and 20+ minute bouts during all hours except 22:00. Sedentary time accumulated in 30+ minute bouts differed between men and women for morning hours (06:00, 07:00, 08:00, 09:00, 10:00 and 11:00) as well as for the 13:00, 14:00, 15:00, 16:00, 17:00, 19:00, 20:00 and 21:00 hours. Gender differences in 60+ minute bouts were observed during the 15:00 and 16:00 hours and for the 20:00, 21:00, and 22:00 hours. For bout lengths of 40+ and 50+, hourly trajectories are shown in the appendix.

## Discussion

Moving from subjective (responses to questions about sitting time and TV watching) to objective (accelerometry) measures of sedentary time has advanced our understanding of sedentary behaviors, yet current analyses using total daily sedentary time may be improved by higher resolution analyses that include assessment of sedentary time patterns. It is particularly important to investigate sedentary time in retirement communities because such settings are critical to healthy aging and quality of life, and are suitable environments for scalable health interventions. By examining sedentary bouts in CCRCs, this study depicted daily *and* hourly patterns of sedentary time and demonstrated how patterns differ among men and women and by age. In terms of total sedentary time (ie, time accumulated in 1+ minute bouts), men were sedentary for more minutes than women independent of age, physical functioning, cognitive functioning, depressive symptoms and accelerometer wear time. Regarding bouts of sedentary time, we observed women had more 1+ minute bouts (i.e., more interruptions to sedentary time) than did men, and with the exception of 5+, 90+ and 120+ minute bouts, women had fewer longer (ie, 10+, 20+, 30+, 40+, 50+, 60+ minute) bouts. We also observed that the largest gender differences in time accumulation occurred in bouts of 10+ and 20+ minutes. Together, this indicates that women accumulate less sedentary time in longer bouts (eg, 10+, 20+) because they break up bouts of sedentary time more often than men. Hourly analyses showed that these gender differences in the number of sedentary time interruptions occurred most often in morning hours.

Improved understanding of sedentary patterns and typical bout lengths can inform intervention strategies. For example, the finding that 58% of older adults’ sedentary time was accumulated in bouts ≥20 minutes suggests that interventions to limit and break up prolonged bouts ≥20 minutes could lead to a more favorable sedentary profile (i.e., less total sedentary time and fewer prolonged bouts, both of which have negative associations with health) [3,13]. These sedentary metrics will also inform epidemiological studies of the health consequences of sitting. Ongoing studies of the negative health effects of prolonged sitting have not yet identified a threshold for how many consecutive minutes one must sit before increasing health risks, but evidence suggests that sitting for as little as 20 consecutive minutes effects cardiometabolic health [13]. Thus, comparing time spent in 20+ minute bouts to total sedentary time may provide a better understanding of the health risks of prolonged sitting.

This study was the first to explore intra-day patterns of sedentary time among older men and women. We observed that as the day progressed, the number of 1+ minute bouts initially increased for both men and women, indicating increased sedentary time interruptions, then around the 09:00 hour decreased over the course of the day. Findings indicated that sedentary time increases later (as compared to earlier) in the day for *both* men and women. The largest gender difference took place in the mornings, when men were more sedentary than women. Evidence from population-based time-use studies indicate that women age 65+ spend more time on household activities (eg, food preparation, housework, and garden/automobile/home maintenance) than men and that the magnitude of the gender differences is higher for adults 75 years and older, which may explain daily gender differences observed in the present study [30]. This increase in gender differences later in life could be due to cultural factors or differences in physical functioning between older men and women. Intra-day allocations of time were not reported in any time-use study including older adults. Future studies should identify if household maintenance is most often done in the early hours of the day because this may account for the observed gender differences in hourly patterns of sedentary time. If patterns of sedentary time are proven to increase health risks, our results justify gender-specific interventions; for example, men may be asked to reduce sedentary time throughout the day, and women in the afternoons and evenings.

Gender differences in sedentary time were also identified in a population-based sample of older Americans [8]. Matthews et al. (2008) observed that men aged 70–85 were sedentary an average of 571.2 minutes daily and women of the same age were sedentary for 546.6 minutes; both estimates are lower than observed in the present study [8]. The large proportion of nonagenarians included in our sample might explain the higher sedentary rates. Given the majority of accelerometer wear time is sedentary, differences in wear time could explain differences in observed sedentary time. The Matthews et al. sample wore devices for an average of 834±114 minutes while our sample wore devices for 812±80.9 minutes. There were no studies of sedentary bouts in *both* older men and women to compare to our total sample. Among men, the number of 5+, 10+, 20+, 30+, 40+, 50+, 60+, 90+, and 120+ minute bouts reported by Jefferis et al. were similar to men in our sample [14]. However, Jefferis et al. reported 71.6 bouts ≥1 minute while only 64.2 bouts of the same length were observed in our sample [14]. Regional and cultural differences might explain the more frequent breaks in sedentary time observed in data from the British Regional Heart Study used by Jefferis et al. Among women, estimates of the number of bouts from our sample were significantly different from those reported Shiroma et al [15]; our sample having fewer 1+, 5+, and 10+ minute bouts and more 20+, 30+, 40+, 50+ and 60+ minute bouts. As in our study, Shiroma et al. observed that age was inversely associated with the number of bouts [15]. The sample in Shiroma et al. was younger than our sample (71.4±5.8 vs. 83.6±6.4 years, respectively) and that may account for the observed differences. Differences in sedentary bouts and in total sedentary time may also result from differences in lifestyle between the general population and those living in CCRCs. CCRCs can provide community engagement activities on campus and also may offer swimming pools, exercise rooms, and common areas for residents to congregate [31]. Context plays a key role in the etiology of all behavior [32] and for sedentary behavior, the social climate and built environment are two key contextual determinants [33]. The present study is the first to describe patterns of sedentary behavior among CCRC residents. Future studies of the etiology of sedentary behavior in older adults should identify the contextual factors specific to CCRCs with a focus on factors that can be modified to improve sedentary profiles among this high risk population.

There are several strengths and limitations that warrant discussion. Our study was conducted with a convenience sample of adults who elected to be involved in a randomized controlled trial and met cognitive and mobility eligibility requirements; they were also predominantly highly educated, white, middle to upper-middle class and lived in CCRCs. Thus, our findings may not generalize to other populations including the broader population of CCRC residents. However, we observed similar gender differences as those observed in a nationally-representative sample, suggesting the gender-difference finding is robust. Furthermore, the sociodemographic homogeneity of our sample allowed for the identification of gender differences in sedentary time and how it was accumulated that were not likely to result from confounders related to race/ethnicity, income, or access to walkable space. Our results are adjusted for several potential confounders of age and gender differences in patterns of sedentary time. Since evidence suggests that suffering from chronic disease is related to age and to patterns of sedentary time [14] future studies should investigate the potential mediating role of chronic diseases in the age-sedentary association. Another limitation is that sedentary time assessed by accelerometry only reflects time when the device was worn. To account for systematic differences related to wear time, we adjusted all models for time spent wearing accelerometers. However, sedentary patterns during device non-wear time are unknown and should be investigated in future studies to better understand 24-hour sedentary patterns. Use of accelerometers to assess sedentary behavior poses challenges because the standard <100 counts per minute cutpoint used to classify sedentary time is an indication of “low movement” that could include standing as well as sitting [34]. While our sample of older adults are unlikely to stand for long periods of time, objective measures that specifically target standing vs. sitting postures such as the activPAL may improve measures of sedentary behavior. New analytic techniques can also be applied to accelerometer data to better predict postures [35]. Finally, in hourly analyses, we used confidence intervals around loess curves to assess statistical significance which is appropriate for the descriptive nature of this study, but is likely a conservative estimate of true hourly gender differences.

In conclusion, our study of sedentary bouts in men and women has demonstrated that in addition to gender differences in total sedentary time, men and women accumulate daily and hourly sedentary time differently. Methods used in this study provide promising new statistical techniques for unpacking total sedentary time into units that may better characterize sedentary behavior [36]. Such techniques will be useful for informing specific behavioral interventions and for studying the health effects of prolonged sitting and of interrupting long bouts of sedentary time. Future studies should investigate the impact of patterns of sedentary time on healthy aging, clinical diseases, and mortality.

## Supporting Information

S1 Fig Number of sedentary bouts started during each one hour period between 06:00 to 22:59. The number of (a) 1+, (b) 5+, (c) 10+, (d) 20+, (e) 30+, (f) 40+, (g) 50+, and (h) 60+ minute bouts are plotted across hours of the day for men and women.
(TIFF)

S2 Fig Sedentary minutes accumulated in bouts of various lengths for each one hour period between 06:00 and 22:59. The number of sedentary minutes spent in bouts of (a) 1+, (b) 5+, (c) 10+, (d) 20+, (e) 30+, (f) 40+, (g) 50+, and (h) 60+ minutes are plotted across hours of the day for men and women.
(TIFF)
