# Supplementary figures and images for: Gender and Age Differences in Hourly and Daily Patterns of Sedentary Time in Older Adults Living in Retirement Communities
Source: PLoS One. 2015 Aug 21;10(8):e0136161. doi: 10.1371/journal.pone.0136161 (PMC4546658; doi:10.1371/journal.pone.0136161)

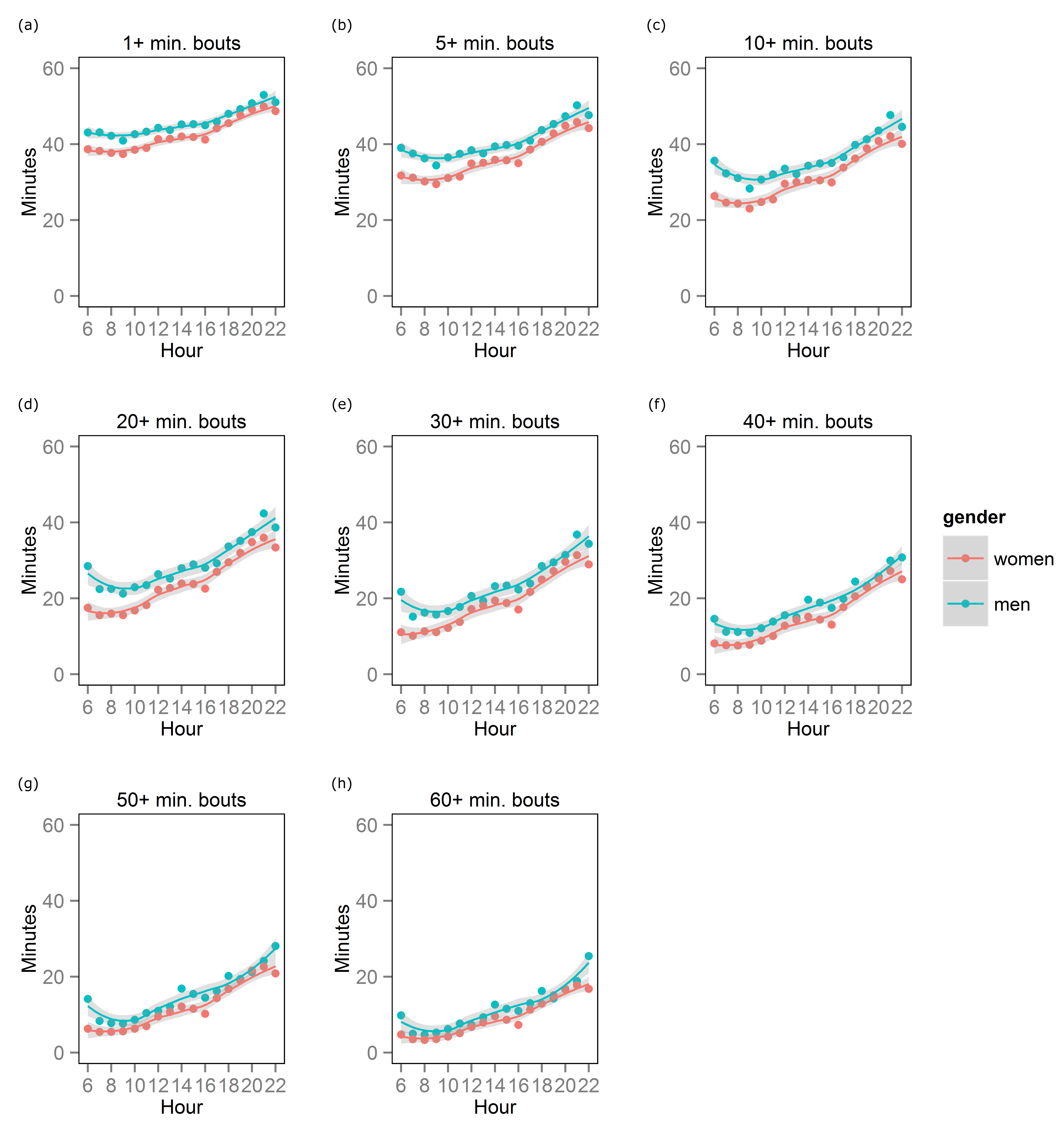

Supplement: S1 Fig — The number of (a) 1+, (b) 5+, (c) 10+, (d) 20+, (e) 30+, (f) 40+, (g) 50+, and (h) 60+ minute bouts are plotted across hours of the day for men and women. (TIFF) [file pone.0136161.s001.tiff]

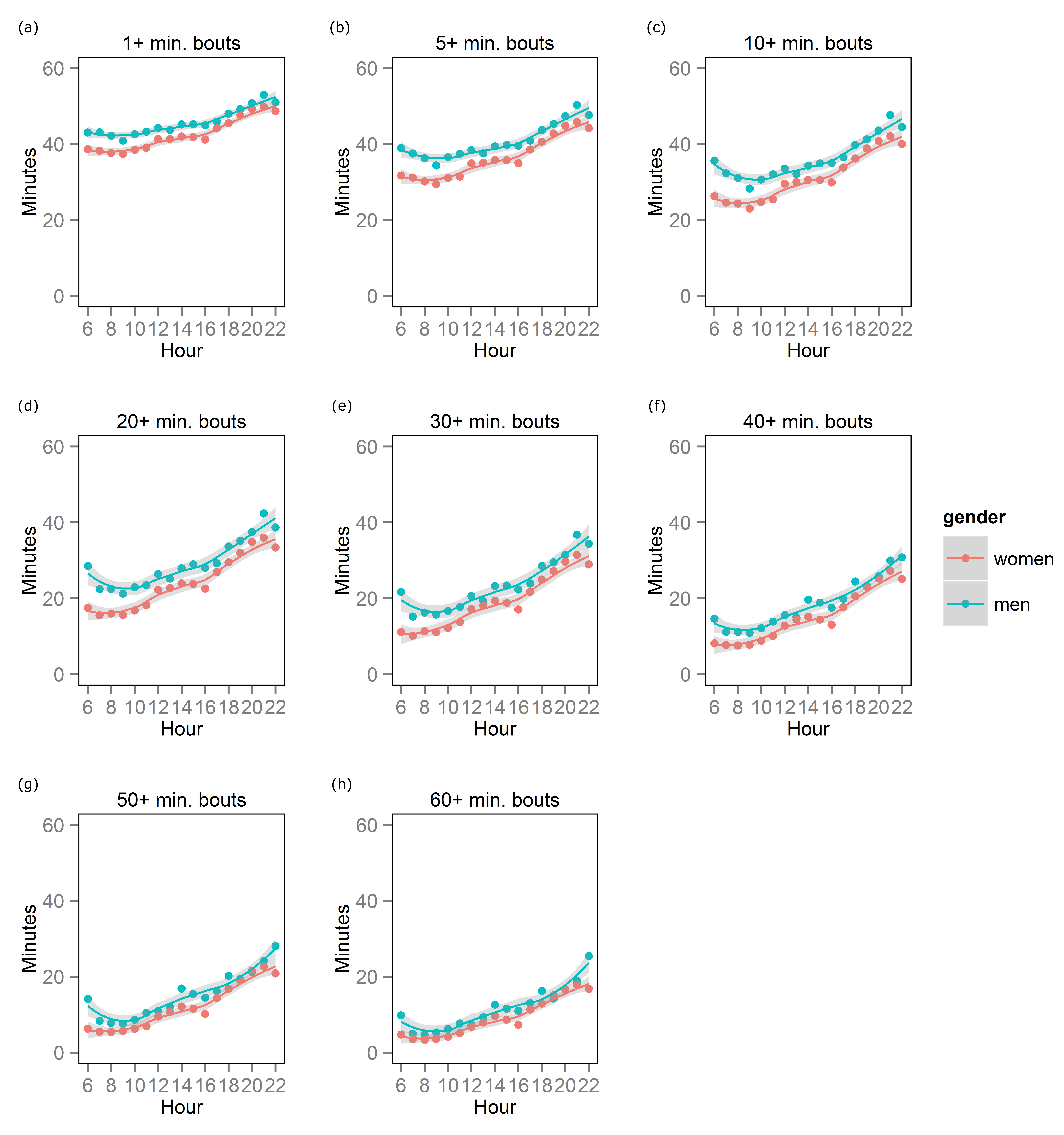

Supplement: S2 Fig — The number of sedentary minutes spent in bouts of (a) 1+, (b) 5+, (c) 10+, (d) 20+, (e) 30+, (f) 40+, (g) 50+, and (h) 60+ minutes are plotted across hours of the day for men and women. (TIFF) [file pone.0136161.s002.tiff]
